# First determination of Pu isotopes (^239^Pu, ^240^Pu and ^241^Pu) in radioactive particles derived from Fukushima Daiichi Nuclear Power Plant accident

**DOI:** 10.1038/s41598-019-48210-4

**Published:** 2019-08-14

**Authors:** Junya Igarashi, Jian Zheng, Zijian Zhang, Kazuhiko Ninomiya, Yukihiko Satou, Miho Fukuda, Youyi Ni, Tatsuo Aono, Atsushi Shinohara

**Affiliations:** 1 Graduate School of Science, Osaka University, 1-1 Machikaneyama, Toyonaka, Osaka 560-0043 Japan; 2 Department of Radioecology and Fukushima Project, National Institutes for Quantum and Radiological Science and Technology, 491 Anagawa, Inage, Chiba 263-8555 Japan; 3 Collaborative Laboratories for Advanced Decommissioning Science, Japan Atomic Energy Agency, 790-1 Otsuka, Motooka, Tomioka, Futaba, Fukushima 979-1151 Japan; 4 State Key Laboratory of Nuclear Physics and Technology, School of Physics, Peking University, Beijing, 100871 China

**Keywords:** Atmospheric chemistry, Geochemistry

## Abstract

Radioactive particles were released into the environment during the Fukushima Dai-ichi Nuclear Power Plant (FDNPP) accident. Many studies have been conducted to elucidate the chemical composition of released radioactive particles in order to understand their formation process. However, whether radioactive particles contain nuclear fuel radionuclides remains to be investigated. Here, we report the first determination of Pu isotopes in radioactive particles. To determine the Pu isotopes (^239^Pu, ^240^Pu and ^241^Pu) in radioactive particles derived from the FDNPP accident which were free from the influence of global fallout, radiochemical analysis and inductively coupled plasma-mass spectrometry measurements were conducted. Radioactive particles derived from unit 1 and unit 2 or 3 were analyzed. For the radioactive particles derived from unit 1, activities of ^239+240^Pu and ^241^Pu were (1.70–7.06) × 10^−5^ Bq and (4.10–8.10) × 10^−3^ Bq, respectively and atom ratios of ^240^Pu/^239^Pu and ^241^Pu/^239^Pu were 0.330–0.415 and 0.162–0.178, respectively. These ratios were consistent with the simulation results from ORIGEN code and measurements from various environmental samples. In contrast, Pu was not detected in the radioactive particles derived from unit 2 or 3. The difference in Pu contents is clear evidence towards different formation processes of radioactive particles, and detailed formation processes can be investigated from Pu analysis.

## Introduction

On 11 March 2011, a major earthquake of magnitude 9 occurred in eastern Japan and resulted in a severe nuclear accident at the Fukushima Daiichi Nuclear Power Plant (FDNPP). This accident was classified at the maximum level of 7 on the International Nuclear and Radiological Event Scale (INES)^1^. Following this accident, large amounts of radionuclides generated in the reactor core were released into the environment through hydrogen explosions in unit 1 and 3, and vent operations and leakages in unit 1, 2, and 3^2^. After 8 years, among the released high volatile radionuclides such as ^129m^Te, ^132^Te, ^131^I, ^133^Xe, ^134^Cs, and ^137^Cs^3–6^, the long-lived radionuclide ^137^Cs (half-life *T*_1/2_ = 30.2 y) still remains and derives a great social influence. Most of the radioactive Cs was released in soluble form such as CsI or CsOH, transported with aerosols, and deposited on soil by wet and dry deposition processes^7^. Among the other long-lived radionuclides, the amounts of ^90^Sr (*T*_1/2_ = 28.7 y) and actinides such as ^239^Pu (*T*_1/2_ = 24100 y), ^240^Pu (*T*_1/2_ = 6560 y) and ^241^Pu (*T*_1/2_ = 14.4 y) have been of concern despite their low volatile property. The amount of ^90^Sr has been quantified and found to be generally lower than that of ^137^Cs in the soil^8,9^.

In a nuclear accident, Pu is one of the most notable radionuclides due to its high radiotoxicity and chemical toxicity^10,11^. The most significant contribution to Pu contamination in the environment is derived from the global fallout due to the nuclear weapon tests during 1945–1980^12^. The other notable contribution is the past nuclear accident in Chernobyl. Contributions from these events are mixed in an environmental sample. To identify the Pu source, the Pu isotopic ratio provides important information because the atom ratio of ^240^Pu/^239^Pu differs according to the Pu source^13^. The ^240^Pu/^239^Pu atom ratio of global fallout was reported as 0.180 ± 0.007^14–16^. On the other hand, in the Chernobyl accident, Muramatsu *et al*.^17^ reported that the ^240^Pu/^239^Pu atom ratio was approximately 0.408 ± 0.003, and higher values of 0.45–0.52 were reported for Chernobyl hot particles^18^; such a high value resulted from the long burn-up time of the nuclear fuel. Before the FDNPP accident, the ratios in the soils ranged from 0.14 to 0.24 in Japan^19,20^ and the Pu contribution mainly originated from global fallout^16^. After the FDNPP accident, the ^240^Pu/^239^Pu atom ratio in various environmental samples around the FDNPP increased compared to the values reported previously. The reported values ranged from 0.286 to 0.381 for the soils, litter, black substances (road dust), vegetation, river water, and air dust samples^21–27^. Nishihara *et al*.^28^ estimated the ^240^Pu/^239^Pu ratio of the fuel inventory in the reactor cores of unit 1–3 as 0.320–0.356 using the ORIGEN2 code and the fuel burn-up data from Tokyo Electric Power Company Holdings, Inc. (TEPCO). The detected ^240^Pu/^239^Pu ratio in the environmental samples were close to that of the fuel inventory calculated by Nishihara *et al*. In addition, the ^241^Pu/^239^Pu atom ratio is also important for identifying Pu source. ^241^Pu/^239^Pu atom ratio of global fallout was reported as 0.00194 ± 0.00014 (^241^Pu decay corrected to 1 January, 2000)^14^. Before the FDNPP accident, the ^241^Pu/^239^Pu atom ratio in fallout materials was 0.00287 ± 0.00028 (^241^Pu decay corrected to 1 January, 2000) in Japan^29^. After the FDNPP accident, a higher value of ^241^Pu/^239^Pu atom ratio (0.103–0.135(^241^Pu decay corrected to 15 March, 2011)) was reported from soil and litter samples around the FDNPP as well as ^240^Pu/^239^Pu atom ratio^21^. However, these environmental samples were influenced by the contamination from the global fallout existing before the FDNPP accident, which hampered the accurate assessment of the environmental impact of the released Pu isotopes.

After the FDNPP accident, small particles containing highly concentrated radioactive Cs (radioactive particles) were detected in various environmental samples such as air dust^30^, soil^31^, vegetation^32^, river water^33^, and dust in residences^34^. The radioactive particles were several micrometres to several hundred micrometres in diameter, mainly comprised of amorphous SiO_2_ and were insoluble in water^30,32^. Previous studies revealed that the radioactive particles also contain Fe, Cr, Mn, Zr, and Zn which were the same as the elemental composition in the structure of the reactor, such as the fuel cladding, vessel, and primary cooling water, and estimated that the radioactive particles were formed in the reactor during the meltdown and directly released from the reactor^35,36^. Physical and chemical properties of radioactive particles play an important role in evaluating the reaction and accident circumstances in a reactor during meltdown^37^. Satou *et al*.^38^ classified the radioactive particles into two types, ‘Type A’ and ‘Type B’, by particle size, Cs concentration, and radioactivity ratio of ^134^Cs/^137^Cs, and estimated that the difference in these types reflected each accident event in the reactor^38^. For ‘Type A’ particles, the diameter is 1–10 µm, Cs is clearly detected by an energy dispersive X-ray spectrometer (EDS), and the ^134^Cs/^137^Cs activity ratio is approximately 1.04^38^. On the other hand, for ‘Type B’ particles, the diameter is 70–400 µm, the Cs concentration is too low to be detected by an EDS, and the ^134^Cs/^137^Cs activity ratio is approximately 0.93^38^. Kogure *et al*.^39^ investigated the distribution of substantial elements, such as Si, O, Cs, Fe, and Zn in ‘Type A’ radioactive particles and revealed that Si, O, Fe, and Zn were homogeneously distributed, while Cs was highly concentrated in the vicinity of the surface. Regarding trace elements in the radioactive particles, the fuel element U was detected in ‘Type A’ by synchrotron radiation (SR) X-ray analysis^35,40^ and secondary ion mass spectrometry (SIMS)^36^, and ^90^Sr was detected in ‘Type B’ by radiochemical analysis^41^. Following the detection of U in ‘Type A’, some studies have suggested that the radioactive particles were formed from the mist which included SiO_2_, Cs, U, and other constituent elements when the molten core dropped on the concrete under the reactor vessel^36,42,43^. In addition, the elemental composition in the radioactive particles are not changed after emission from the reactor due to their insolubility in water. The chemical property of the radioactive particles was stable in the environment and retained the original property at the time of emission from the reactor. In addition, the radioactive particles were free from the influence of the global fallout. Therefore, the amount of Pu in radioactive particles is important to estimate the detailed formation process of radioactive particles in the reactor, for example, the reaction temperature condition.

In this study, we perform radiochemical analysis to determine the amount of Pu and the atom ratios of ^240^Pu/^239^Pu and ^241^Pu/^239^Pu for the radioactive particles from the FDNPP accident. In addition, the formation process of radioactive particles in the reactor is discussed based on the amount of Pu.

## Methods

### Sampling locations

Two environmental samples were collected near the FDNPP. One is a bulk dust sample collected on 23 January 2015 from an open garage building in Futaba town, Fukushima Prefecture, 3 km north-west of the FDNPP (37°44′N and 141°02′E). The other is a bulk soil sample collected on 1 July 2016 in Okuma town, Fukushima Prefecture, 3 km south-south-west of the FDNPP (37°40′N and 141°02′E). Details of the sample collection were described in our previous study^38,41^.

### Extraction and isolation of a radioactive particle

We obtained the radioactivity distributions in the dust and soil samples by autoradiography using an imaging plate (IP) (BAS-SR 2025, FUJIFILM Corporation., Japan) sensitive to β-rays and gamma-rays to identify the radioactive particles from these samples. The IP was set on the sample with an exposure time of 10 min and high radioactivity spots were identified. By separating the spots in the bulk samples and repeating the IP measurements, we reduced the volume of the samples. Then, the radioactive particles were isolated from the environmental samples by the multiple wet separation method developed by Miura *et al*.^33^. Finally, 1 mL water containing only a radioactive particle was obtained and the solution was dried on a carbon tape of size 2 mm × 2 mm. The shape and major elements of the radioactive particle were identified and measured by scanning electron microscopy (SEM) (VE-9800, Keyence Inc., Japan) equipped with an EDS (EDAX, AMETEK Co., Ltd., USA). In the analysis, acceleration voltage of the scanning electron beam was set to 20 keV.

### Gamma-ray measurement

The gamma-rays from the radioactive particles attached to the carbon tape were measured by a germanium semiconductor detector (GEM40-76, ORTEC, USA). The activities of ^134^Cs and ^137^Cs were measured by the gamma-ray intensities of 604 keV and 662 keV, respectively. The detection efficiencies of the gamma-rays from the radioactive particles were determined by a standard sample quantified at University of Tsukuba^38,41^.

### Radiochemical analysis for quantifying Sr and Pu in the radioactive particles

In this study, we quantified ^90^Sr in the radioactive particles before quantifying Pu. Strontium-90 in the radioactive particles was purified and concentrated by solid-phase extraction and was measured by liquid scintillation counter^41^. In the first step, the radioactive particle was dissolved by alkali fusion. The radioactive particle with the carbon tape was put into a zirconium crucible, and 2 mL of high purity 3 M NaOH solution (Kanto Chemical Co., Inc., Japan) was added. The crucible was heated to 80 °C in an electric furnace to evaporate the solution, and then it was further heated to 350 °C for 30 min. After cooling, the residue in the crucible was dissolved by 90 mL of 3 M HNO_3_ solution. The solution was passed through the Sr Rad Disk (3 M, Inc., USA) to trap the Sr. The passed solution was later used for Pu identification. The trapped Sr was eluted from the Disk using a 0.02 M EDTA solution. The solution was then passed through the ion-exchange resin followed by 2% (w/v) EDTA solution to remove natural radionuclide of ^210^Pb which would interfere with β-ray measurement from the resin. The ^90^Sr fraction eluted by 3 M HCl solution from the resin was set into the liquid scintillation counter (1220 Quantulus, Perkin Elmer Inc.). Cherenkov light from ^90^Y, a daughter nuclide of ^90^Sr fraction was continuously measured over 2 weeks. Detailed procedures are described in Zhang *et al*.^41,44^.

**Figure 1:**
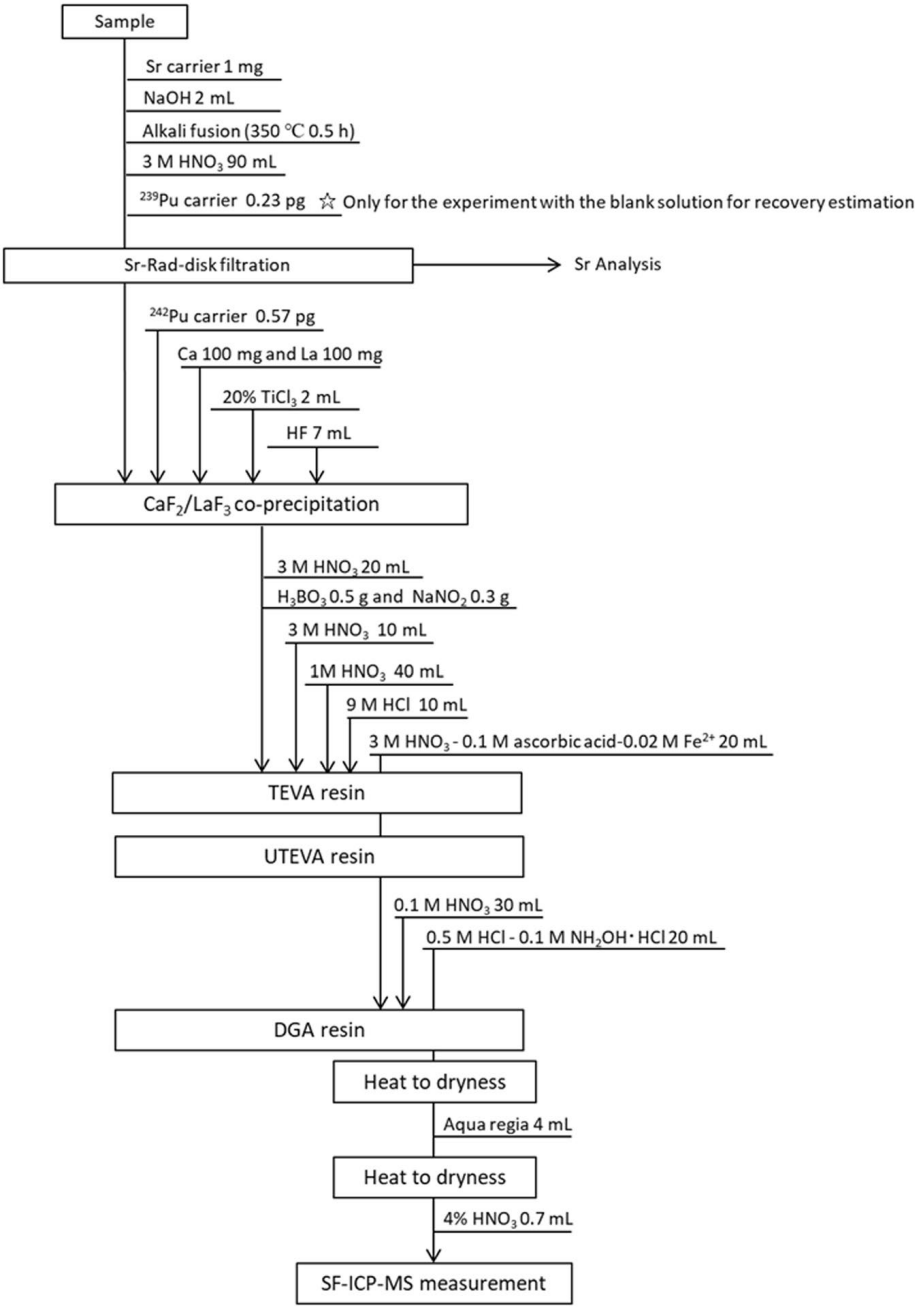
Procedure of radiochemical analysis for quantifying Pu in radioactive particles.

To quantify Pu in the radioactive particle, we performed radiochemical analysis to purify and concentrate Pu and measured Pu isotopes by inductively coupled plasma-mass spectroscopy (ICP-MS). As a chemical yield carrier, 0.57 pg of ^242^Pu was added to the solution passing through the Sr Rad Disk. To adjust Pu to Pu (III) state which undergoes better co-precipitation during the following CaF_2_/LaF_3_ co-precipitation procedure, 2 mL of 20% TiCl_3_ solution was added to the sample solution. Following the addition of 100 mg Ca and 100 mg La, 7 mL HF was pipetted into the sample solution and the centrifuge tube was shaken to facilitate the co-precipitation. After allowing 10 min for the reaction, the precipitate was separated from the supernatant by centrifugation at 3000 rpm for 15 min. The supernatant was discarded, and the precipitate was dissolved in 20 mL of 3 M HNO_3_ with the addition of 0.5 g H_3_BO_3_. Finally, 0.3 g NaNO_2_ was added to the sample solution for valence adjustment of Pu to the tetravalent state by heating at 40 °C for 30 min. This solution was ready for the following extraction chromatographic separation. An extraction chromatography was performed with a combination of TEVA, UTEVA, and DGA resin cartridges (2 mL each, Eichrom Technologies, Inc., USA) to separate Pu from the interfering elements, such as U, Pb, Tl, Bi and Th^45^. After the chromatographic separation, the purified Pu solution was evaporated to dryness at 200 °C, and the residue was dissolved in 4 mL aqua regia and heated to dryness again. Finally, the sample residue was dissolved in 0.7 mL of 4% HNO_3_ solution for sector field (SF)-ICP-MS measurement. The whole procedure of Pu separation is shown in Fig. 1, and further step-by-step details were described by Wang *et al*.^45^.

### Estimating total chemical yield of quantifying Sr and Pu in the radioactive particle

In the Pu quantification process, chemical yield of Pu was identified by ^242^Pu carrier added for passed solution of the Sr-Rad Disk. The Pu loss by the Sr-Rad Disk was investigated to estimate the total chemical yield. For this purpose, we prepared a blank solution (3 M HNO_3_ solution) with 0.23 pg of ^239^Pu and performed the same radiochemical separation as that for radioactive particle Pu analysis.

### SF-ICP-MS measurement

SF-ICP-MS (Element XR, Thermo Fisher Scientific, Germany) was used to detect Pu. The APEX-Q sample introduction system (Elemental Scientific, USA) was combined with SF-ICP-MS to improve the sensitivity and suppress the formation of UH^+^ and UH_2_^+^, which interfere with the accurate Pu measurement. The amount of Pu isotopes was determined from the mass region of 239, 240 and 241. The mass region of 242 was also measured to obtain the chemical yield from the spiked ^242^Pu. From these measurements, the radioactivities of ^239+240^Pu and ^241^Pu, and atom ratios of ^240^Pu/^239^Pu and ^241^Pu/^239^Pu were determined. The detailed settings of the measurement were reported by Zheng *et al*.^46^.

## Results

### Shape and radioactivity of the radioactive particle

**Figure 2:**
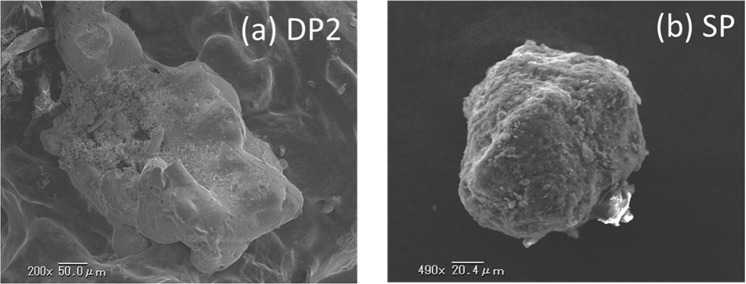
SEM images of the radioactive particles (**a**) DP2 and (**b**) SP.

**Figure 3:**
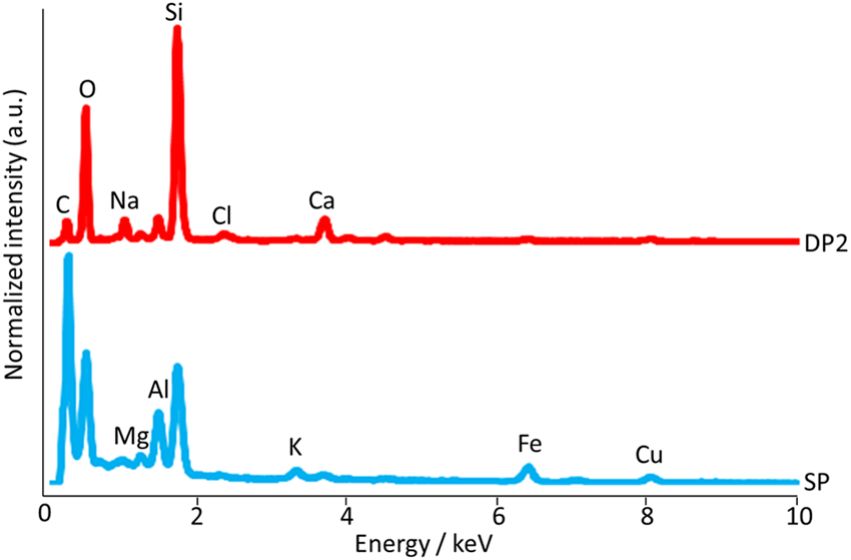
EDS spectra of the radioactive particles of DP2 and SP.

Three radioactive particles named DPs were obtained from the dust samples, and one radioactive particle named SP was obtained from soil samples. Figure 2 shows the SEM images of representative DPs (DP2) and SP. The SEM images of other particles are shown in Fig. S1. The diameter of DPs were several hundred micrometers. For the SP, the diameter was determined to be 120 µm from the SEM image. Figure 3 shows the EDS spectra of representative DPs (DP2) and SP. The EDS spectra of other particles were shown in Fig. S2. From these spectra, for the whole of the particles, it is observed that DPs and SP were mainly composed of SiO_2_. Moreover, the Cs peak was not clearly observed. The ^137^Cs radioactivity of these radioactive particles identified from gamma-ray spectrometry was 234 ± 1 Bq, 887 ± 4 Bq, 736 ± 4 Bq and 224 ± 1 Bq for DP1, DP2, DP3 and SP, respectively on 11 March 2011. The ^134^Cs/^137^Cs activity ratios of DPs ranging from 0.895 ± 0.005 to 0.922 ± 0.006 were clearly different from that of SP, 1.03 ± 0.01 as shown in Table 1.

**Table 1:** Activities of ^134^Cs,^137^Cs, ^90^Sr, ^239+240^Pu and ^241^Pu, atom ratio of ^240^Pu/^239^Pu and ^241^Pu/^239^Pu and radioactivity ratio of ^134^Cs/^137^Cs, ^90^Sr/^137^Cs, ^239+240^Pu/^137^Cs and ^241^Pu/^239+240^Pu for the analysed radioactive particles (DPs and SP).

Radioactive particle		DP1	DP2	DP3	SP
Activity [Bq]	^134^Cs	214 ± 2	794 ± 4	679 ± 4	230 ± 1
	^137^Cs	234 ± 1	887 ± 4	736 ± 4	224 ± 1
	^90^Sr	0.171 ± 0.010	0.589 ± 0.034	Not measured^*^	0.0455 ± 0.0040
	^239+240^Pu	(1.70 ± 0.20) × 10^−5^	(3.48 ± 0.45) × 10^−5^	(7.06 ± 0.57) × 10^−5^	<4.44 × 10^−6^
	^241^Pu	<1.97 × 10^−3^	(4.10 ± 0.68) × 10^−3^	(8.10 ± 1.27) × 10^−3^	<2.23 × 10^−3^
Atom ratio	^240^Pu/^239^Pu	0.330 ± 0.077	0.415 ± 0.069	0.373 ± 0.045	—
	^241^Pu/^239^Pu	—	0.178 ± 0.016	0.162 ± 0.028	—
Radioactivity ratio	^134^Cs/^137^Cs	0.915 ± 0.009	0.895 ± 0.005	0.922 ± 0.006	1.03 ± 0.01
	^90^Sr/^137^Cs	(7.31 ± 0.43) × 10^−4^	(6.64 ± 0.39) × 10^−4^	—	(2.03 ± 0.18) × 10^−4^
	^239+240^Pu/^137^Cs	(7.26 ± 0.86) × 10^−8^	(3.92 ± 0.51) × 10^−8^	(9.58 ± 0.78) × 10^−8^	<1.98 × 10^−8^

^241^Pu decay was corrected to 11 March, 2011. The activities of ^90^Sr are quoted from Zhang *et al*.^41^. The analytical error is based on 1σ counting statics. *This radioactive particle was not analysed for ^90^Sr in the previous study^41^.

### Quantifying the amount of Pu in the radioactive particle

The yield of radiochemical analysis of Pu for each sample obtained from the ^242^Pu amount added after Sr separation was 91.0 ± 5.0%, 92.9 ± 5.3%, 95.5 ± 5.7% and 89.5 ± 3.9% for DP1, DP2, DP3 and SP, respectively. The total chemical yield including Sr separation method was determined from the analysis of operational blank sample with ^239^Pu spike as 92.3 ± 2.8%. Therefore, the loss of Pu in Sr separation was negligible.

Figure S3 shows ICP-MS spectra for radioactive particles and operational blank sample. The counting rates in the mass region of 239 (^239^Pu) and 240 (^240^Pu) were 166 and 66 cps, 289 and 121 cps and 938 and 357 cps, for DP1, DP2 and DP3, respectively. These rates were much higher than those for the operational blank sample (less than 15 cps), thereby indicating that we successfully identified the Pu contained in radioactive particles for the first time. The results of quantifying Pu in the radioactive particles are summarized Table 1. The radioactivity of ^239+240^Pu was (1.70 ± 0.20) × 10^−5^ Bq, (3.48 ± 0.45) × 10^−5^ Bq and (7.06 ± 0.57) × 10^−5^ Bq for DP1, DP2 and DP3, respectively. The ^240^Pu/^239^Pu atom ratio was 0.330 ± 0.077, 0.415 ± 0.069 and 0.373 ± 0.045 for DP1, DP2 and DP3, respectively. On the other hand, the counting rates for the SP sample were 39 and 18 cps for ^239^Pu and ^240^Pu, respectively. These rates were just slightly higher than those for the operational blank sample, thereby limiting an accurate quantification of Pu isotopes. Nevertheless, an upper limit of ^239+240^Pu activity contained in SP was estimated as 4.44 × 10^−6^ Bq. In addition, ^241^Pu was also quantified from counting rates in the mass region of 241 for DP2 and DP3. The radioactivity of ^241^Pu was (4.10 ± 0.68) × 10^−3^ Bq and (8.10 ± 1.27) × 10^−3^ Bq, for DP2 and DP3 respectively and the ^241^Pu/^239^Pu atom ratio was 0.178 ± 0.016 and 0.162 ± 0.028 for DP2 and DP3, respectively on 11 March 2011. On the other hand, the counting rates of ^241^Pu for DP1 and SP were very close to the operational blank sample limiting its accurate quantification. We could estimate an upper limit of ^241^Pu as 1.97 × 10^−3^ Bq and 2.32 × 10^−3^ Bq, for DP1 and SP, respectively.

## Discussion

Different activity ratios of radioactive Cs were detected for DPs and SP (Table 1). Using the ORIGEN code, Nishihara *et al*.^28^ calculated the ^134^Cs/^137^Cs activity ratio as 0.95, 1.08, 1.05 for the units 1, 2, and 3, respectively. The ^134^Cs/^137^Cs activity ratios of DPs and SP were compared with the calculated values, and we estimated that DPs were derived from unit 1 and SP was derived from unit 2 or 3. DPs were identified as ‘Type B’ due to the similarity in particle size, Cs concentration, and ^134^Cs/^137^Cs activity ratio, according to the classification by Satou *et al*.^38^. SP was similar to ‘Type B’ with respect to the particle size and Cs concentration. However, with respect to the ^134^Cs/^137^Cs activity ratio, SP was similar to ‘Type A’ rather than ‘Type B’. We conclude that SP had differences with the features of both ‘Type A’ and ‘Type B’ and was classified as a new type of radioactive particle^41^.

**Figure 4:**
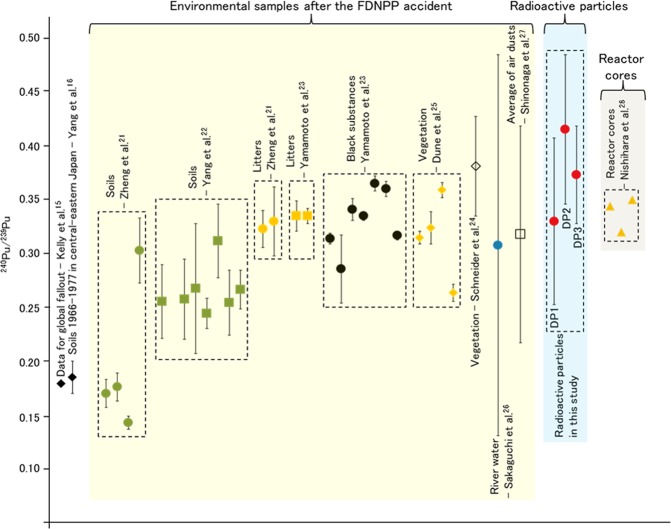
Comparison of atom ratios of ^240^Pu/^239^Pu in various environmental samples, radioactive particles, and source terms: global fallout^15^, soils during 1966–1977 in Japan^16^, soil after the FDNPP accident^21,22^, litter^21,23^, black substance^23^, vegetation^24,25^, river water^26^, average of air dusts^27^, and calculated result of reactor core^28^.

**Figure 5:**
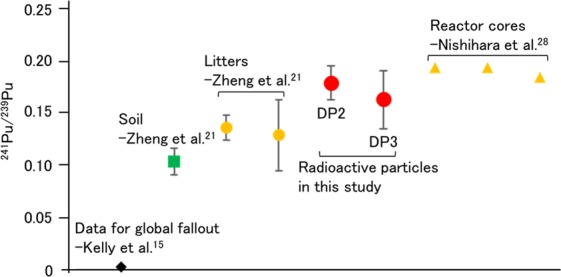
Comparison of atom ratios of ^241^Pu/^239^Pu in various environmental samples, radioactive particles, and source terms: global fallout^15^, soil and litter after the FDNPP accident^21^, and calculated result of reactor core^28^.

The ^240^Pu/^239^Pu and ^241^Pu/^239^Pu atom ratios in the radioactive particles in this study were compared with the value calculated using ORIGEN code. The ^240^Pu/^239^Pu ratios in units 1, 2, and 3 reactor cores were 0.344, 0.320, and 0.350 calculated from fuel inventories by Nishihara *et al*.^28^. These ratios of DPs (DP1: 0.330 ± 0.077, DP2: 0.415 ± 0.069, DP3: 0.373 ± 0.045) generally agreed well with the calculated values of each reactor. The ^241^Pu/^239^Pu ratios in units 1, 2, and 3 reactor cores were 0.192, 0.192, and 0.183 also calculated by Nishihara *et al*.^28^. These ratios of DPs (DP2: 0.178 ± 0.016, DP3: 0.162 ± 0.028) are also consistent with these calculated values of each reactor. However, due to the relatively large error in the measured values, we cannot distinguish the source unit of the radioactive particles only from these atom ratios. The obtained ^240^Pu/^239^Pu and ^241^Pu/^239^Pu atom ratios in the radioactive particle were also compared with those of various environmental samples, such as soil^21,22^, litter^21,23^, black substance^23^, vegetation^24,25^, river water^26^, and air dust^27^, as shown in Figs 4 and 5. The ^240^Pu/^239^Pu atom ratio detected in environmental samples ranged from 0.14 to 0.381. In particular, the ratios (the numbers in brackets) in litters obtained by Yamamoto *et al*. (0.335 ± 0.007)^23^ and Zheng *et al*. (0.323 ± 0.017–0.330 ± 0.032)^21^, in black substances reported by Yamamoto *et al*. (0.335 ± 0.004–0.365 ± 0.015)^23^, and in vegetable reported by Dunne *et al*. (0.324 ± 0.015 – 0.359 ± 0.07)^25^ showed relatively high values among them. It was inferred that these samples were less contaminated by the global fallout source of Pu due to the extremely low transfer factor of global fallout Pu from soil to plant^47^. The ratios in DPs were consistent with the ratios in these environmental samples. In contrast, the values of ^240^Pu/^239^Pu atom ratio in soils reported by Zheng *et al*.^21^ and Yang *et al*.^22^ were lower than that of DPs. The ^241^Pu/^239^Pu atom ratios in DPs agreed better with litters obtained by Zheng *et al*. (0.128 ± 0.034–0.135 ± 0.012)^21^ than soil obtained by Zheng *et al*. (0.103 ± 0.013)^21^ as well as the ^240^Pu/^239^Pu atom ratio. As pointed out by previous studies^21,22^, these environmental samples were strongly influenced by global fallout, the value of which was 0.180 ± 0.007 for ^240^Pu/^239^Pu atom ratio^14–16^ and 0.00194 ± 00014 for ^241^Pu/^239^Pu atom ratio^14^. The Pu contribution from the FDNPP accident can be estimated using the two end-member model^48^, and the extent of Pu contribution in soil samples was estimated as follows: 87% by Zheng *et al*.^21^ and 31–59% by Shibahara *et al*.^49^. In the radioactive particle, the Pu injection occurred only in the reactor unlike other environmental samples. Thus, radioactive particle samples are completely free from the influence of the global fallout and the ^240^Pu/^239^Pu and ^241^Pu/^239^Pu atom ratio reflect the ratio in the core of the FDNPP directly. Therefore, both atom ratio of radioactive particles could provide us more accurate information on the ratio in the core of the FDNPP than other environmental samples and has great potential for source identification of the released radioactive particles in the environment, which should be further explored in a future study.

**Figure 6:**
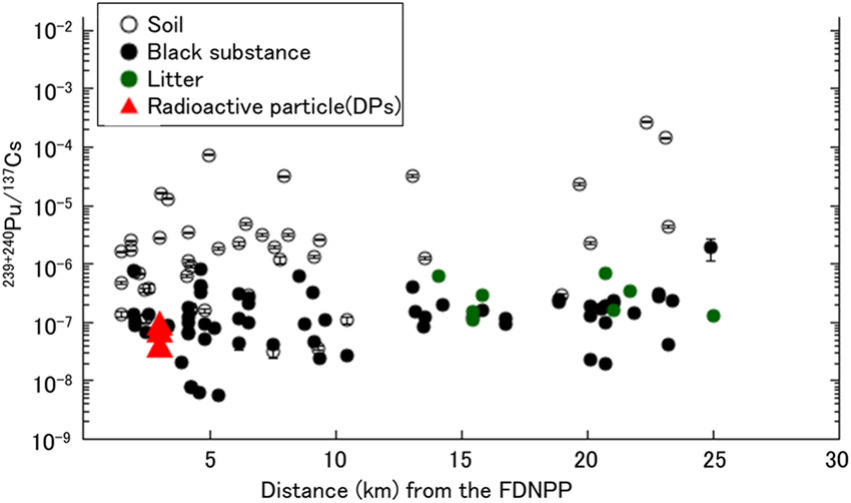
Relationship between the activity ratios of ^239+240^Pu/^137^Cs and the distance to sampling site from the FDNPP within 25 km around the FDNPP. (Data of soils, black substances are quoted from Yamamoto *et al*.^23^, and data of litters are quoted from Yamamoto *et al*.^23^ and Zheng *et al*.^21^).

Figure 6 shows the relationship between the activity ratio of ^239+240^Pu/^137^Cs and the distance to the sampling site from the FDNPP. The ^239+240^Pu/^137^Cs activity ratio of DPs was (7.26 ± 0.86) × 10^−8^, (3.92 ± 0.51) × 10^−8^ and (9.58 ± 0.78) × 10^−8^ for DP1, DP2 and DP3, respectively. These ratios were two orders of magnitude smaller than those in soils reported by Yamamoto *et al*.^23^. In contrast, these values agreed well with those in black substances and litters reported by Yamamoto *et al*.^23^ and Zheng *et al*.^21^, respectively. This result is consistent with the discussion on ^240^Pu/^239^Pu and ^241^Pu/^239^Pu atom ratio. The typical value of ^239+240^Pu/^137^Cs activity ratio of the global fallout was of the order of 10^−3^ ^50^. Therefore, we conclude that the soil samples were strongly influenced by the global fallout compared with the black substances and litters as mentioned in the discussion on ^240^Pu/^239^Pu and ^241^Pu/^239^Pu atom ratio. The relationship between the ^239+240^Pu/^137^Cs activity ratio and the distance from the FDNPP is important for understanding the dispersion and deposition behavior of the volatile element such as Cs and non-volatile elements such as Pu in the environment. The ^239+240^Pu/^137^Cs activity ratios in black substances and litters agreed with those in the radioactive particles, although the distance from the FDNPP were completely different. The ratios of radioactive particles directly reflected the released ratio of Pu to Cs from the reactor. The agreement indicates that the behavior of dispersion and deposition of Pu in the environment after being released from the reactor was similar to that of Cs within a distance of 25 km, as pointed out by Yamamoto *et al*.^23^.

The determination of Pu isotopes contained in the radioactive particles can reveal not only the accurate ^240^Pu/^239^Pu and ^241^Pu/^239^Pu atom ratio derived from the FDNPP accident but also provide insight into the formation process of the radioactive particles. In case of the Chernobyl accident, ‘hot particles’ containing U and other actinides were released. Lancsaris *et al*.^51^ reported that the ^239+240^Pu activities of the hot particles were in the range (4.2–92) × 10^−3^ Bq. On the other hand, the ^239+240^Pu activities of the radioactive particles released from the FDNPP accident were very small ((1.70–7.06) × 10^−5^ Bq) compared to that of the hot particles released from the accident in Chernobyl. Such a difference in Pu activity among the particles could be attributed to the formation process of these particles in the reactor between the radioactive and hot particle. The hot particles were considered to be originating from the fragments of the fuel core, and the particles were formed during the explosive break-up of the reactors^52^. In the case of radioactive particles released from the FDNPP accident, however, a previous study related to the formation process of the ‘Type B’ (which is of the same type as DPs) indicated that fission product gases were incorporated into the silicate-rich materials such as glass fiber insulator materials surrounding the reactor pressure vessel^53^. Our first identification of Pu in the radioactive particles indicated that fuel elements such as Pu were introduced to the silicate materials at the time of the formation of ‘Type B’ radioactive particles. Aberecht *et al*.^54^ estimated that refractory species having the least oxygen, such as Pu_2_O_3_, were evaporated by the reductive reaction in the reductive atmosphere of the reactor due to the large amounts of hydrogen gas during the meltdown. During the formation process of ‘Type B’ radioactive particle, we estimated that the mist, including the evaporated Pu_2_O_3_ and other fission products such as Cs, were incorporated into the insulator materials in the reductive atmosphere. The contents of the mist should change with rising temperature of the mist according to the volatile pressure of each element. Therefore, investigating the amounts of various elements contained in the radioactive particles enabled us to understand the detailed formation process of the particles. The ^239+240^Pu/^137^Cs activity ratio was different for DPs ((3.92–9.58) × 10^−8^) and SP (<1.98 × 10^−8^), which implies that the formation processes of both were different. We estimated that the temperature in unit 1 was higher than that in unit 2 or 3 during the formation of the radioactive particles.

Similar to Pu, Sr is one of the trace elements contained in the radioactive particles. Zhang *et al*.^41^ reported that the activity ratio of ^90^Sr/^137^Cs in radioactive particles was of the order of 10^−4^. The activities of ^90^Sr were 0.171 ± 0.010 Bq, 0.589 ± 0.034 Bq and 0.0455 ± 0.0040 Bq for DP1, DP2 and SP, respectively. The relationship between DPs and SP for the ^90^Sr/^137^Cs activity ratio and ^90^Sr activity was similar, as summarised in Table 1. Pontillon *et al*.^55^ and Lewis *et al*.^56^ observed that the release fraction from the core inventory of Sr was much lower than that of Cs and that of Pu was even lower. Therefore, we considered that the amount of Pu in the radioactive particles, which is less volatile than Sr, would be clearly different between DPs and SP due to the rising fuel temperature in the core. Pontillon *et al*.^55^ also categorised Ag as a volatile element along with Cs and showed that the emission of Ag increased with the rise in temperature, while Cs was released from the fuel pellet until 2300 K. Satou^57^ considered that the release rate of ^110m^Ag reflected the fuel temperature in the core and investigated the activity ratio of ^110m^Ag/^137^Cs in soils. From the result of his investigation, he estimated that the temperature of unit 1 was higher than that of unit 2 or 3 when the radionuclides were released into the environment, which is characterized by the high ^110m^Ag/^137^Cs activity ratio. Therefore, we estimated that DPs derived from unit 1 were formed under a high temperature atmosphere, where non-volatile radionuclides such as Pu were more volatile compared to SP derived from unit 2 or 3.

In this study, we first investigated the amounts of Pu in DP (Type B) radioactive particles. On the other hand, Pu was not detected from radioactive particles that were not categorized as ‘Type A’ or ‘Type B’. The amount of Pu in ‘Type A’ particles still remains to be investigated. The formation process of radioactive particles can be investigated by measuring Pu amount and comparing between different particle types. In this study, the error of obtained Pu isotopic ratio was relatively large because we quantified trace amounts of Pu in the radioactive particles. To obtain more accurate ratio, it is necessary to quantify Pu from the Pu-bearing particles that contain more concentrates Pu than the radioactive particles. For this purpose, we consider investigating the Pu-bearing particles and quantifying Pu in these particles by identification methods such as the alpha particle imaging detector developed by Morishita *et al*.^58^ in the future.

## Supplementary information

First determination of Pu isotopes (<sup>239</sup>Pu, <sup>240</sup>Pu and <sup>241</sup>Pu) in radioactive particles derived from Fukushima Daiichi Nuclear Power Plant accident

## Data Availability

The data that support the findings of this study are available upon request from the corresponding author [JI].
