# Supplementary material for: First determination of Pu isotopes (239Pu, 240Pu and 241Pu) in radioactive particles derived from Fukushima Daiichi Nuclear Power Plant accident
Source: Sci Rep. 2019 Aug 14;9:11807. doi: 10.1038/s41598-019-48210-4 (PMC6694128; doi:10.1038/s41598-019-48210-4)
Supplement: Supplementary file 1 — First determination of Pu isotopes (239Pu, 240Pu and 241Pu) in radioactive particles derived from Fukushima Daiichi Nuclear Power Plant accident [file 41598_2019_48210_MOESM1_ESM.pdf]

SUPPORTING INFORMATION

First determination of Pu isotopes (^{239}Pu, ^{240}Pu and ^{241}Pu) in radioactive particles derived from Fukushima Daiichi Nuclear Power Plant accident

Junya Igarashi[1 *], Jian Zheng[2 *], Zijian Zhang[1], Kazuhiko Ninomiya[1], Yukihiko Satou[3], Miho Fukuda[2], Youyi Ni[2,4], Tatsuo Aono[2] & Atsushi Shinohara[1]

[1]Graduate School of Science, Osaka University, 1-1 Machikaneyama, Toyonaka, Osaka 560-0043, Japan.

[2]Department of Radioecology and Fukushima Project, National Institutes for Quantum and Radiological Science and Technology, 491 Anagawa, Inage, Chiba 263-8555, Japan.

[3]Collaborative Laboratories for Advanced Decommissioning Science, Japan Atomic Energy Agency, 790-1 Otsuka, Motooka, Tomioka, Futaba, Fukushima 979-1151, Japan.

[4]State Key Laboratory of Nuclear Physics and Technology, School of Physics, Peking University, Beijing, 100871, China.

[*]Correspondence and request for materials should be addressed to J.I. (email:igarashij17@chem.sci.osaka-u.ac.jp) or J.Z. (email: zheng.jian@qst.go.jp).

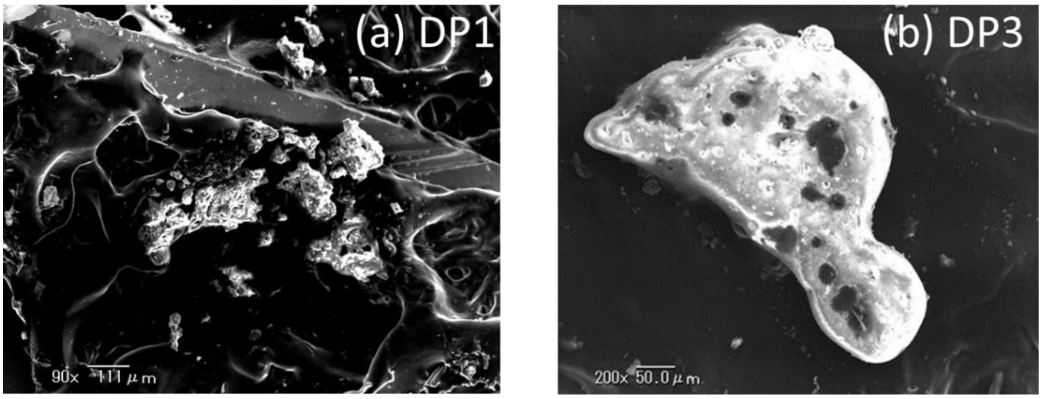

Figure S1. SEM images of the radioactive particles (a) DP1 and (b) DP3

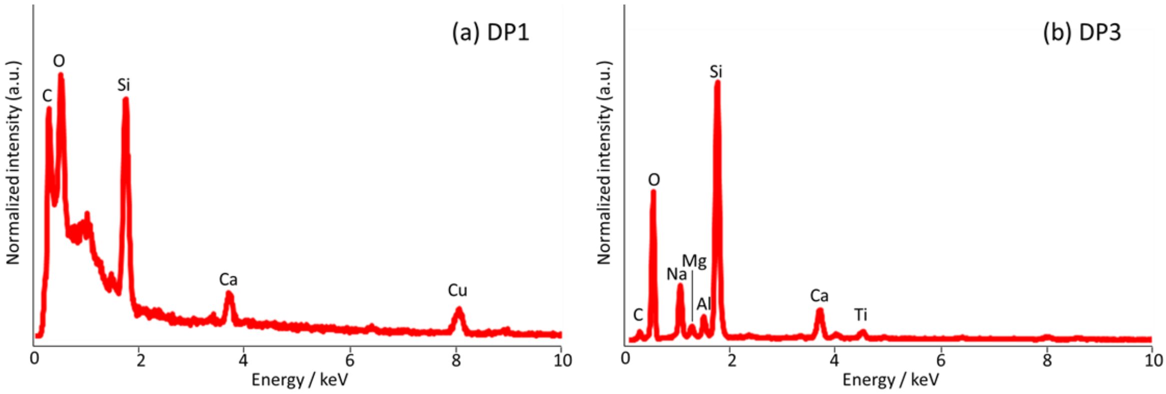

Figure S2. EDS spectra of the radioactive particles (a) DP1 and (b) DP3

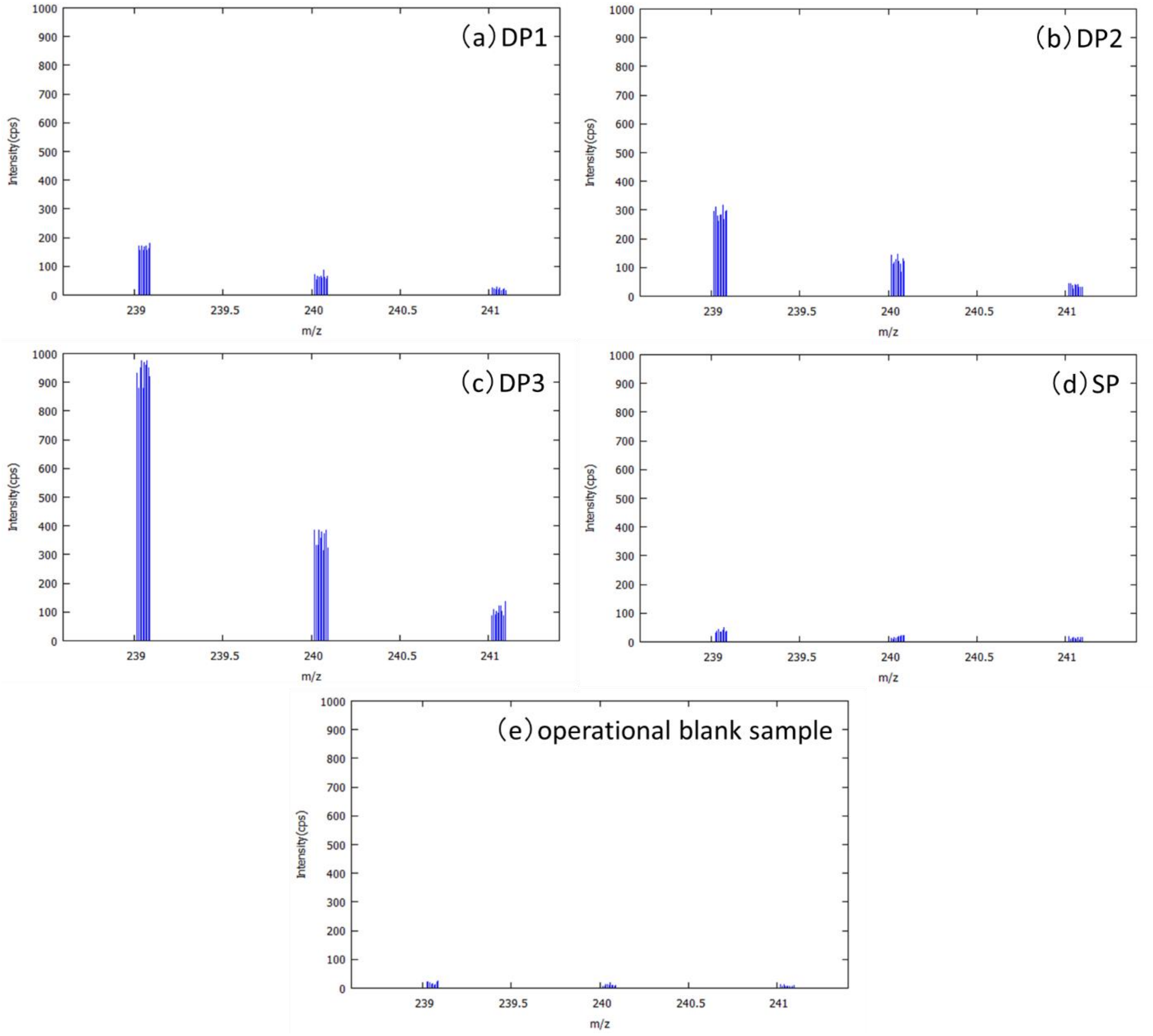

Figure S3. Spectra for SF-ICP-MS measurement of Pu isotopes for (a) DP1, (b)DP2, (c)DP3, (d) SP, and (e) operational blank sample
